# Longitudinal Changes in Whole-Brain Functional Connectivity Strength Patterns and the Relationship With the Global Cognitive Decline in Older Adults

**DOI:** 10.3389/fnagi.2020.00071

**Published:** 2020-03-17

**Authors:** Qiongge Li, Chao Dong, Tao Liu, Xiaodan Chen, Alistair Perry, Jiyang Jiang, Jian Cheng, Haijun Niu, Nicole A. Kochan, Henry Brodaty, Perminder S. Sachdev, Wei Wen

**Affiliations:** ^1^School of Biological Science and Medical Engineering, Beihang University, Beijing, China; ^2^Centre for Healthy Brain Aging, School of Psychiatry, University of New South Wales, Sydney, NSW, Australia; ^3^Beijing Advanced Innovation Center for Big Data-Based Precision Medicine, Beihang University, Beijing, China; ^4^Beijing Advanced Innovation Center for Biomedical Engineering, Beihang University, Beijing, China; ^5^National Key Laboratory of Cognitive Neuroscience and Learning, Beijing Normal University, Beijing, China; ^6^Beijing Key Laboratory of Brain Imaging and Connectomics, Beijing Normal University, Beijing, China; ^7^Max Planck UCL Centre for Computational Psychiatry and Aging Research, Berlin, Germany; ^8^Neuropsychiatric Institute, Prince of Wales Hospital, Sydney, NSW, Australia; ^9^Dementia Centre for Research Collaboration, School of Psychiatry, University of New South Wales, Sydney, NSW, Australia

**Keywords:** aging, functional connectivity strength, global cognition, longitudinal changes, resting-state fMRI

## Abstract

Aging is associated with changes in brain functional patterns as well as cognition. The present research sought to investigate longitudinal changes in whole brain functional connectivity strength (FCS) and cognitive performance scores in very old cognitively unimpaired individuals. We studied 34 cognitively normal elderly individuals at both baseline and 4-year follow-up (baseline age = 78 ± 3.14 years) with resting-state functional magnetic resonance imaging (r-fMRI), structural MRI scans, and neuropsychological assessments conducted. Voxel-based whole brain FCS was calculated and we found that bilateral superior parietal and medial frontal regions showed decreased FCS, while the supplementary motor area (SMA) and insula showed increased FCS with age, along with a decrease in bilateral prefrontal cortical thickness. The changes of FCS in left precuneus were associated with an aging-related decline in global cognition. Taken together, our results suggest changes in FCS with aging with the precuneus as a hub and this may underlie changes in global cognition that accompany aging. These findings help better understand the normal aging mechanism.

## Introduction

Normal aging has been associated with cognitive decline, affecting various cognitive domains such as processing speed, memory, and executive function (Hedden and Gabrieli, 2004; Fjell et al., 2015, 2017). Cognitive performance which may change with age is associated with communication between brain regions, as well as extrinsic interactions between functional networks (Fox et al., 2005; Onoda et al., 2012; Fjell et al., 2016; Perry et al., 2017). However, there are few resting-state functional magnetic resonance imaging (r-fMRI) studies investigating how age-related brain functional connectivity and their relationships with cognition evolve over time in cognitively unimpaired older adults.

Most r-fMRI studies have reported that aging-related functional connectivity decrements preferentially affect the default mode network (DMN) (Damoiseaux et al., 2008; Koch et al., 2010; Wang et al., 2010; Tomasi and Volkow, 2012) and the dorsal attentional network (DAN; Tomasi and Volkow, 2012) (for a comprehensive review, see Ferreira and Busatto, 2013). Previous longitudinal studies about functional connectivity have brought new insights into aging (Fjell et al., 2016; Salami et al., 2016). A longitudinal study found that inter-network functional connectivity between executive control network and DMN showed a U-shaped trajectory, initially increasing over time and later decreasing as participants aged (Ng et al., 2016). A recent study also found that whole-brain and DMN functional connectivity exhibited a non-linear trajectory (Staffaroni et al., 2018). However, most of the previous aging-related studies explored the aging brain by comparing resting-state functional connectivity, few have compared whole brain functional connectivity strength (FCS) which can permit a comprehensive characterization of the functional properties of each voxel (Boccaletti et al., 2006; Rubinov and Sporns, 2010). FCS is computed by summing weights of all the connections of a given voxel that exceeded a predefined optimized threshold, and it is also known as weighted degree centrality (Buckner et al., 2009). Brain regions with relatively high FCS can be regarded as functional hubs in large-scale brain networks (Buckner et al., 2009; Tomasi and Volkow, 2011; Dai et al., 2015). Furthermore, the whole brain voxel-based FCS applied in the current study can enable whole brain hub mapping but avoid parcellation-dependency (Smith et al., 2011). To the best of our knowledge, no study has yet investigated the longitudinal changes in whole brain FCS of cognitively unimpaired older individuals.

Longitudinal studies have indicated that decreased functional connectivity within DMN, particularly the precuneus/posterior cingulate cortex (PCC), was correlated with memory declines (Persson et al., 2014; Bernard et al., 2015), episodic memory, and processing speed changes (Staffaroni et al., 2018). However, the relationship between longitudinal changes of whole brain FCS and longitudinal changes of cognitive performance scores, especially global cognition, in very old cognitively unimpaired people are poorly understood. In addition, a longitudinal study over a mean follow-up interval of 8 years found that age-related decline in cortical thickness is widespread (Thambisetty et al., 2010). A recent study highlights that cortical thickness changes should be considered when evaluating the standard deviation (SD) of the BOLD signal alternations in the lifespan (Pur et al., 2019). Therefore, investigation about longitudinal FCS and cognition combined with longitudinal cortical thickness may be a useful tool to detect perturbations in brain function and structure in cognitively normal subjects.

In the current study, first, we sought to determine the FCS patterns at two time points and whether there are significant FCS changes between two time points. We then sought to verify whether the observed changes in FCS are influenced by gray matter volume. Second, we aimed to find whether 4-year FCS changes are related to the significantly decreased cognitive domains. In addition, we aimed to explore whether there are cortical thickness changes in 4-year’s follow-up, which can help to understand the brain-aging mechanism.

## Materials and Methods

### Participants

Participants were drawn from the Sydney Memory and Aging Study (MAS), a longitudinal study of non-demented, community-dwelling individuals aged 70–90 years old at baseline (Sachdev et al., 2010). MAS participants were recruited randomly from the eastern suburbs of Sydney, Australia, using the electoral roll, for which registration is compulsory for Australian citizens. At baseline, each of 1037 MAS participants was administered a comprehensive neuropsychological test battery, and 542 (52.3%) also had an MRI scan. Individuals were excluded if they had a Mini-Mental State Examination (MMSE) score < 24 (Folstein et al., 1975; Anderson et al., 2007), a diagnosis of dementia, mental retardation, psychotic disorder (including schizophrenia and bipolar disorder), multiple sclerosis, motor neuron disease, progressive malignancy, or inadequate English to complete assessments. Participants were classified as cognitively unimpaired if performance on all test measures was above the 6.68 percentile (−1.5 SDs) or equivalent score compared to normative published values, they were not demented, and they had normal function or minimal impairment in IADLs defined by a total average score < 3.0 on the Bayer ADL scale (Hindmarch et al., 1998). All the participants were first examined in 2005–2007, and re-examined approximately 2, 4, 6 years later, and fMRI scans were performed at 2 and 6 years in a proportion of participants. For the current study, we used these two time points as baseline and 4-year follow-up assessments. Details of the sampling methodology have been published previously (Sachdev et al., 2010). The study was approved by the Ethics Committees of the University of New South Wales and the South Eastern Sydney and Illawarra Area Health Service. Written informed consent was obtained from each participant.

For the current study, we only included participants who were classified as cognitively unimpaired from neuropsychological assessments and received r-fMRI scans at both baseline and 4-year follow-up (*N* = 38). All 38 participants had an MMSE score higher than 26. After excluding participants from non-English speaking background (NESB) (Anderson et al., 2007), imaging artifacts (including the metal artifact), and damaged image, 34 participants were finally included in the current study (demographic characteristics in Table 1).

**TABLE 1:** Demographic and cognitive characteristics for the study sample.

**Variable**	**Baseline**	**4-year follow-up**	***P*-value**
Age (mean ± SD, years)	78.27 ± 3.14	82.19 ± 3.17	<0.001*
Gender (M/F)	14/20	14/20	–
Education (mean ± SD, years)	12.54 ± 2.90	12.54 ± 2.90	–
MMSE (mean ± SD)	28.91 ± 1.00	28.82 ± 1.00	0.702

*Significant difference between baseline and 4-year follow-up. MMSE: Mini-Mental State Examination.

### Neuropsychological Assessments

All eligible participants (*N* = 34) received assessments for a comprehensive neuropsychological battery (Sachdev et al., 2010). Five cognitive domains were tested, including processing speed, executive function, language, visuo-spatial, and memory (Supplementary Table S1). Specially, executive function in the present study includes the Controlled Oral Word Association Test (FAS) and Trail Making Test (TMT) B (Supplementary Table S1). FAS measures letter-based word retrieval, adherence to rule constraints. TMT B measures complex attention, set-shifting, psychomotor speed, and visual search. Raw component test scores were transformed to z scores based on sample means and SDs [z = (raw – mean)/SD], and domain composite scores were calculated by averaging z scores of component tests. Global cognition score was calculated for each participant as the average of all composite domain z scores. The signs of z scores of TMT A and TMT B were reversed, so for all composites of cognitive domains, greater positive scores represented better performance. Paired *t*-test was performed to compare the cognitive scores changes between baseline and 4-year follow-up. IBM SPSS Statistics 21 was used to analyze the behavioral data, and data were screened for univariate outliers.

### Image Acquisition

Magnetic resonance imaging scans were conduct on a Philips 3T Achieva Quasar Dual scanner (Philips Medical Systems, Best, Netherlands), at NeuRA (Neuroscience Research Australia), Sydney. All participants were instructed to keep their eyes closed and think nothing when receiving resting-state fMRI scans. A T2^∗^-weighted echo planar imaging (EPI) sequence was used, with the following parameters: repetition time (TR)/echo time (TE) = 2000/30 ms, flip angle = 90°, field of view (FOV) = 240 × 130.5 × 240 mm^3^, 29 continuous axial slices, slice thickness = 4.5 mm without interslice gap, matrix size = 128 × 128, resulting in voxel size = 1.9 × 1.9 × 4.5 mm^3^. During the 7-min scan of fMRI, we required 208 volumes per subject. A 3D T1-weighted structural MRI was acquire d during the same scanning session with TR/TE = 6.39/2.9 ms, flip angle = 8°, FOV = 256 × 256 × 190 mm^3^, slice thickness = 1.0 without interslice gap, resulting an isotropic voxel size = 1 × 1 × 1 mm^3^.

### Images Processing

All the fMRI data were pre-processed and analyzed using DPABI (Yan and Zang, 2010), which is a toolbox for data processing based on Statistical Parametric Mapping (SPM12). The first 10 volumes were discarded to allow the magnetization to approach a dynamic equilibrium and for the participants to get used to the scanner noise. R-fMRI data were then corrected for slice-timing to the median reference slice and realigned for head motion correction. No subject was excluded under a head motion criterion of 3 mm and 3°. We then normalized all images to Montreal Neurological Institute (MNI) space by using EPI template and resliced with a voxel size of 3 mm × 3 mm × 3 mm to agree with the gray matter probability maps. Spatial smoothing was then applied with a 4-mm full-width half-maximum (FWHM) Gaussian Kernel. Several nuisance variables, including Friston’s 24 head motion parameters (Friston et al., 1996), the averaged signal from white matter and cerebrospinal fluid tissue, were removed through multiple linear regression analysis to reduce the effects of non-neuronal signals (Fox et al., 2005). The SPM-defined cerebrospinal fluid and white matter masks were used to extract the mean signal for nuisance regression. Finally, a bandpass filter (0.01–0.1 Hz) was applied to reduce the very low frequency and high frequency noise.

### FCS Estimates

Whole-brain FCS analysis was performed. First, the blood oxygen level-dependent (BOLD) time-series of each pair of voxels within a gray matter mask without cerebellum (with the number of voxels of the mask *N* = 45381) was calculated. The mask was generated by selecting a threshold of 0.2 on the gray matter probability map (>0.2) provided by SPM12 and extracting overlapping voxels in the automated anatomical labeling template (Tzourio-Mazoyer et al., 2002). Then only Pearson’s correlation coefficients above a threshold of *r* > 0.2 were used, because the physiological basis of the negative correlations was ambiguous (Fox et al., 2009). And this threshold was typically used in previous study (Liao et al., 2013). Next, the degree strength of each voxel was defined as D(*i*) = ∑*r*_*ij*_,*where*j = 1…*N*,*i*≠*j*. Finally, the values of the degree map were standardized to z scores to make them comparable across subjects:

$$\text{FCS}\,\left(i\right)=\frac{D_{i}-u}{\delta },\,1\leq i\leq N$$

where *u* and δ are mean and SD of the degree strength across all *N* nodes.

This high threshold was chosen to eliminate counting voxels that had low temporal correlation attributable to signal noise. Different threshold selections did not qualitatively change the results for cortex (see Supplementary Figures S1, S2). The FCS voxel-wise analysis and voxel-wise covariates were performed using DPABI and MATLAB code in-house.

### Analysis of FCS Patterns at Two Time Points

We estimated the FCS patterns at this two time points before longitudinal analysis. One-sample *t*-test was performed to explore the FCS patterns at each time point (two-tailed GRF correction, voxel-wise: minimum z-value > 3.29; cluster significance: *p* < 0.05). The peak coordinates and assignation of Brodmann’s area labels were performed using SPM12.

### Analysis of Longitudinal Changes in FCS

To explore the 4-year changes in age-related changes of FCS, we defined longitudinal FCS changes as follow-up minus baseline (ΔFCS). The statistical significance of differences in whole brain degree maps between baseline and 4-year follow-up were examined with 10,000 random permutations and corrected for multiple comparisons [cluster-defining threshold: *p* ≤ 0.01, family-wise error (FWE) corrected: *p* ≤ 0.05]. The permutation test was performed using Statistical Non-Parametric Mapping (SnPM)—a toolbox for SPM (Nichols and Holmes, 2014).

### Validation Test: Longitudinal Changes in FCS With Gray Matter Volume as Additional Covariates

In order to estimate whether these results are robust, we performed this validation test. Given the controversy surrounding the idea that differences in the functional connectivity might result from a structural abnormality in gray matter volume (He et al., 2007; Oakes et al., 2007; Kathirvelu et al., 2019), we performed the analysis of voxel-based global cortical gray matter volume in SPM 12. We applied a voxel-based morphometry (VBM) protocol using the DPABI in SPM12. Gray matter, white matter, and cerebrospinal fluid were segmented on each subject’s individual T1-weighted sequence using SPM12. We derived each subject’s gray matter volume map at two time points fromT1 structural images using the longitudinal pre-processing pipeline. FCS differences between baseline and 4-year follow-up were re-examined using paired *t*-test by taking individual gray matter volume of two time points as additional covariates (Supplementary Figure S3). The analyses were carried out with multiple comparisons correction (two-tailed GRF correction, voxel-wise: minimum z-value > 3.29; cluster significance: *p* < 0.05). The Statistical Analysis sub-toolbox of DPABI was used to perform a paired *t*-test with gray matter volume as a voxel-wise covariate.

### Correlation Between Changes in FCS and Changes in Cognitive Performance

At each time point, we computed the relationship between FCS and three cognitive domains which showed significantly decline in 4 years (processing speed, executive function, and global cognition), with sex, baseline age, and gray matter volume reduction as covariates. Furthermore, in order to investigate the FCS and cognition associations in longitudinal settings, we also focused on 4-year changes of FCS and the significantly decreased cognitive domain scores (processing speed, executive function, and global cognition). Decreased scores were computed as follow-up minus baseline (Δcognition). Changes in FCS were also computed as follow-up minus baseline (ΔFCS). Pearson’s correlation was used to assess the relationship between ΔFCS and Δcognition, with sex, baseline age, and gray matter volume reduction as covariates. Multiple comparisons were carried out by two-tailed Gaussian random field (GRF) theory correction (voxel-wise: minimum z-value > 3.29; cluster significance: *p* < 0.05).

### Cortical Thickness Measurement

To measure cortical thickness, T1-weighted images were processed with the longitudinal pipeline available in FreeSurfer (Reuter et al., 2012). Specifically, this pipeline creates an unbiased within-subject template space and image using robust, inverse consistent registration (Reuter and Fischl, 2011). The following processing steps were also included: skull stripping, Talairach transforms, atlas registration, spherical surface maps, and parcellation of cerebral cortex. Based on gyral and sulcal anatomy, the cortex was segmented into 34 different gyral and sulcal anatomy using the Desikan–Killiany Atlas (Desikan et al., 2006). For each of these regions, mean cortical thickness was calculated as the distance (in mm) between the pial and gray/white matter surfaces. FreeSurfer results were manually checked for segmentation and registration accuracy. Four-year changes in cortical thickness were investigated by vertex-based paired *t*-test obtained with general linear models (GLMs) using the QDEC toolbox in FreeSurfer [corrected with false discovery rate (FDR) < 0.05].

Even though cortical thickness may be more sensitive in explaining variability in the BOLD signal compared to GM volume, it is impossible to control for cortical thickness in a voxel-wise analysis as it is vertex-based. Therefore, to control for connectivity differences due to GM differences, VBM was used. However, since differences in the thickness of the cortical mantle may be the main driver of differences in GM volume, age-related changes in cortical thickness were also examined.

## Results

### Neuropsychological Tests

The neuropsychological results are summarized in Table 2. Significant decline was found in global cognition and two cognitive domains (processing speed, executive function) in 4 years.

**TABLE 2:** Cognitive domain scores.

**Cognitive domain**	**Baseline**	**4-year follow-up**	***P*-value**
Processing speed	0.60 ± 0.65	0.16 ± 1.05	0.003*
Executive function	0.64 ± 0.79	0.44 ± 0.85	0.019*
Language	0.40 ± 0.76	0.42 ± 0.83	0.816
Visuo-spatial	0.35 ± 0.88	0.30 ± 0.95	0.648
Memory	0.74 ± 0.87	0.74 ± 0.81	0.983
Global cognition	0.72 ± 0.69	0.54 ± 0.88	0.023*

*Difference between baseline and 4-year follow-up is significant (*P* < 0.05). Data in first two columns are means ± standard deviations.

### FCS Patterns at Two Time Points

**FIGURE 1:**
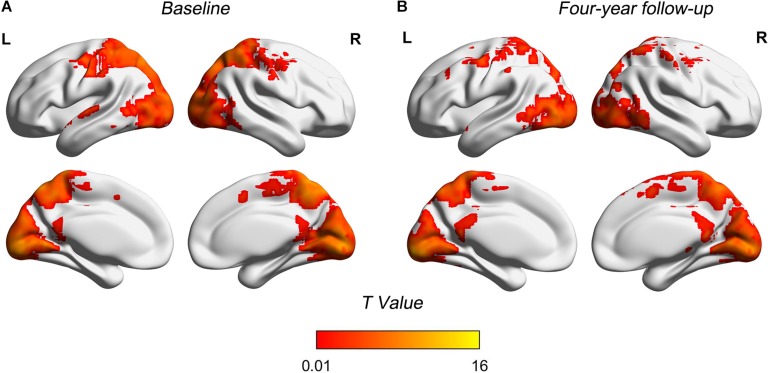
Spatial FCS patterns of two time points. **(A)** FCS patterns in baseline. **(B)** FCS patterns in 4-year follow-up. Brain regions showing higher FCS were mainly located in precuneus, calcarine, inferior occipital, and inferior temporal. FCS maps at two time points showed similar distribution patterns. FCS, functional connectivity strength; T, statistical value; L, left bran; R, right brain.

Figure 1 shows patterns of distributions of FCS at both baseline and follow-up, highlighting the similarity of the patterns at the two time points (Table 3). Main clusters were located in bilateral precuneus, left calcarine, left inferior occipital, and right inferior temporal. The regions with strong connections to other brain regions were approximately bilaterally symmetrical.

**TABLE 3:** FCS patterns at two time points.

				**Peak MNI**			
				**coordinates,**			
				**mm**			
			**Cluster**				
	**Brain regions**	**BA**	**size**	***x***	***y***	***z***	**T**
Baseline	Left precuneus	7	10,405	–6	–63	63	15.79
	Right precuneus	5	–	3	–54	51	14.63
	Right precuneus	7	–	6	–69	57	13.02
4-year	Left calcarine	18	9694	–9	–96	–6	14.86
follow-up	Left inferior occipital	19	–	–51	–69	–15	12.96
	Right inferior temporal	37	–	51	–60	–21	11.60

*BA, Brodmann’s area; MNI, Montreal Neurological Institute; T, statistical value of peak voxel; All listed clusters significant at *p* < 0.01.*

### Longitudinal Changes in FCS

**FIGURE 2:**
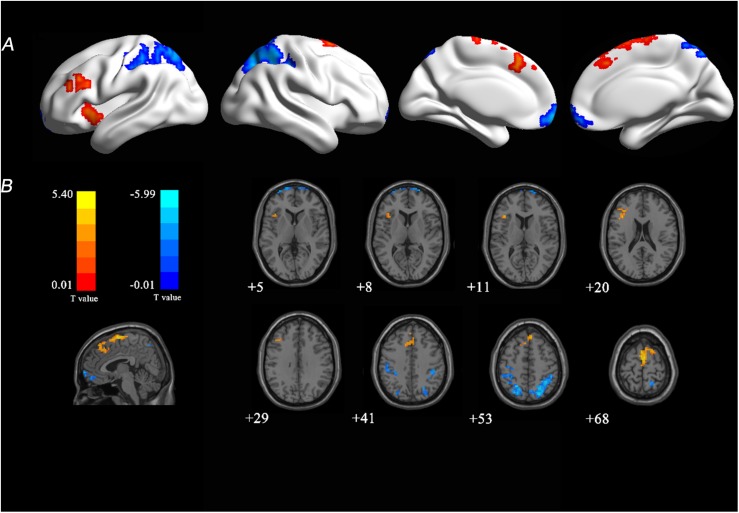
T-statistical FCS map at 4-year follow-up compared with baseline. **(A)** Longitudinal changes were overlaid on three-dimensional standard brain. **(B)** Longitudinal changes were overlaid on two-dimensional standard brain. Warm colors (red and yellow) represent increased FCS and cool colors (blue) represent decreased FCS with age. FCS, functional connectivity strength; T, statistical value.

There were significant FCS changes in five cerebral regions when comparing baseline with follow-up (Figure 2). Left supplementary motor area (SMA) (BA 6) and left insula (BA 13) showed increased FCS over time. Decreased FCS was observed in the right medial frontal (BA 11) and bilateral superior parietal lobes (BA 7) (10,000 random permutations for two-sample *t*-test, cluster-defining threshold: *p* ≤ 0.01, FWE-corrected: *p* ≤ 0.05). The detailed information for each cluster was summarized in Table 4. Moreover, in the validation test, our main findings were preserved after correcting for gray matter volume (Supplementary Figure S3).

**TABLE 4:** Regions showing significant differences in FCS with normal aging.

				**Peak MNI**			
				**coordinates,**			
				**mm**			
			**Cluster**				
	**Brain regions**	**BA**	**size**	***x***	***y***	***z***	**T**
Increased	Left SMA	6	339	–6	–3	78	5.40
FCS	Left insula	13	189	–39	18	–6	4.16
Decreased	Right superior parietal	7	395	21	–72	54	5.12
FCS	Left superior parietal	7	320	–24	–57	54	5.39
	Right medial frontal	11	284	12	69	–3	5.99

*BA, Brodmann’s area; MNI, Montreal Neurological Institute; FCS, functional connectivity strength; T, statistical value of peak voxel; SMA, supplementary motor area; All listed clusters significant at *p* < 0.01.*

### Association Between ΔFCS and ΔCognition

**FIGURE 3:**
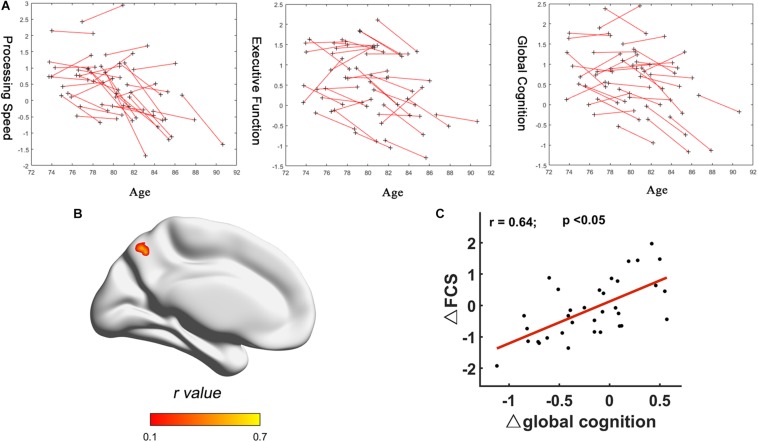
Correlation between FCS changes and cognition changes. **(A)** Longitudinal trajectories of cognitive data: processing speed, executive function, and global cognition. **(B)** R-statistical map of association between ΔFCS and Δglobal cognition. **(C)** Scatter plot of fitted ΔFCS of peak voxel in the left precuneus versus Δglobal cognition. Each data point represents a single subject. FCS, functional connectivity strength.

No significant correlation between FCS and cognitive domains (processing speed, executive function, and global cognition) was found at each time point. But significant positive correlation between Δglobal cognition and ΔFCS (two-tailed GRF correction, voxel-wise: minimum z-value > 3.29; cluster significance: *p* < 0.05) was found in the left precuneus (Figure 3). No significant relationship was found between Δprocessing speed and ΔFCS, or Δexecutive function cognition and ΔFCS. The peak location of relationship between ΔFCS and Δglobal cognition was then used to define the seed region to further explore the connectivity patterns in the sample (Supplementary Material).

### Longitudinal Changes in Cortical Thickness

**FIGURE 4:**
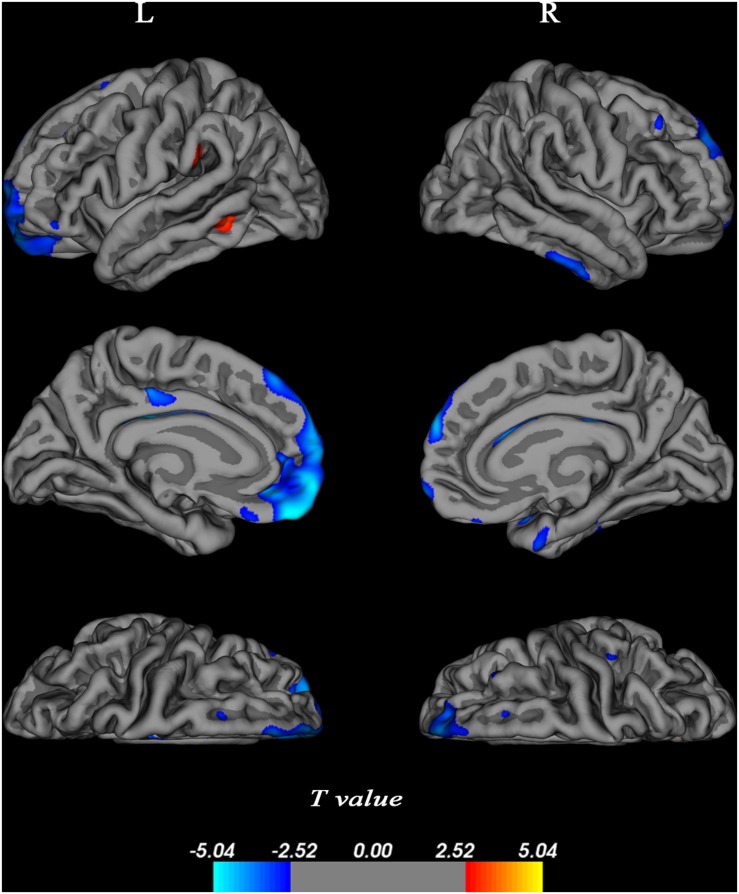
Changes in cortical thickness over 4 years. Cortical thickness was determined using a general linear model corrected at a false discovery rate (FDR) of 0.05 and projected onto a semi-inflated brain. Decreased cortical thickness was indicated in blue, and increased cortical thickness was presented in red. L, left bran; R, right brain.

Longitudinal assessment of changes in cortical thickness revealed cortical thinning over large areas of bilateral frontal (BA 9/10/11), small area of left middle cingulum (BA 23), and part of right temporal cortex (BA 20/36) (*p* < 0.05, FDR-corrected, Figure 4). No significant associations were found between alterations of cortical thickness and cognitive domain scores.

## Discussion

In this longitudinal study, we explored the changes of FCS, cortical thickness, and cognitive performance over a period of 4 years in cognitively normal old people. Most observed longitudinal FCS changes remained after correcting for longitudinal reduction in gray matter volume. In addition, the positive relationship between 4-year changes of FCS and aging-related decline in global cognition was found in left precuneus, which was then used as a seed to investigate its functional connectivity with other voxels in the brain.

Aging-related functional connectivity decrements preferentially affect DMN and the DAN (Ferreira and Busatto, 2013). In this study, we found that decreased FCS was located primarily in the DMN and DAN regions, i.e., right medial frontal and bilateral superior parietal, consistent with previous study (Tomasi and Volkow, 2012). The DMN, which has been the main focus of aging-related fMRI research, comprises a set of cortical regions that include the medial prefrontal cortex, the inferior parietal lobule, the hippocampus, and precuneus (Raichle et al., 2001; Ferreira and Busatto, 2013). Furthermore, the DMN regions, the core components of functional hubs, are involved in a variety of functional processes. The medial prefrontal cortex shows high metabolic activity at rest (Raichle et al., 2001). Medial frontal cortex was shown to play a critical role in performance monitoring and the implementation of associated adjustments in cognitive control (Ridderinkhof et al., 2004). Superior parietal regions correlate with episodic retrieval and the phenomenological experience of remembering (Wagner et al., 2005). According to our findings, the FCS in those regions decreased with aging, which may affect some related functions. Tomasi and Volkow (2012) also found that aging was associated with decreased functional connectivity density, similar to FCS in the present study, in DMN. Taken together, these findings provide the longitudinal evidence that the influence of advancing age is mainly in right medial frontal and bilateral superior parietal, evidenced by disturbance of FCS in these areas.

Increased FCS with normal aging was observed in the left insula and left SMA in our study. Cauda et al. (2011) investigated functional connectivity of insula in the resting brain and reported the role of insula in sensorimotor integration and links to SMA. These findings may partly explain the synchronous changes of FCS in these two areas. A previous study found that long-range functional connectivity density in insula, somatosensory, and motor cortex increased with age (Tomasi and Volkow, 2012). Insula and SMA are related to the salience network which regulates dynamic changes in other networks (Bonnelle et al., 2012) and participates in the integration of sensory data (Seeley et al., 2007). There is growing evidence to suggest that the integrity of the salience network is necessary for the efficient regulation of activity in the DMN (Bonnelle et al., 2012). As for the findings of increased FCS in the present study, one possible explanation is that it is a compensatory increase. Another interpretation is that aging-related dedifferentiation processes (Cabeza et al., 2002) or neural strategies change in the brain. Age-related increases may reflect the use of alternative cognitive strategies with aging, or they may signal beneficial compensatory activity (Cabeza et al., 1997). These altered FCS patterns may interact with each other to keep the participants still holding a cognitively normal state across four years. The evidence for possible compensatory mechanisms is important and would also be well suited for future studies. Intriguingly, parietal and SMA not only showed relatively high FCS (Figure 1), but also exhibited altered FCS (Figure 2), which may be taken to support the hypothesis that highly connected regions would be particularly vulnerable to aging effects, related to its central role as mechanisms subtending lifetime brain plasticity (Fjell et al., 2014).

In the current study, global cognition and two of five cognitive performances (processing speed and executive function) decreased significantly over 4 years. Intriguingly, no significant correlation between FCS and these three cognitive domains (processing speed, executive function, and global cognition) was found at each time point. Only the positive relationship between Δglobal cognition and ΔFCS was found in the left precuneus. These interesting findings indicated that correlation between ΔFCS and Δglobal cognition was more significant than any correlation between FCS and cognitive scores at each time point. Previous studies have suggested that the precuneus, which is a core hub in DMN, plays an important role in a diverse array of highly integrated functions (Leech and Sharp, 2014) and is relevant for episodic memory retrieval (Greicius et al., 2004). Longitudinal studies have indicated that memory declines are related to decreased functional connectivity within DMN, particularly the precuneus/PCC (Persson et al., 2014; Bernard et al., 2015). The positive relationships indicated that more the FCS decreased in precuneus, greater the decline in global function with aging. The relationship between FCS changes and cognition changes was observed for global cognition rather than the individual domains, possibly because of the likely role of the FCS in integrating multiple cognitive functions, and the greater sensitivity of the global cognitive measure. Only one region, the precuneus, showed a significant relationship between change in brain connectivity and change in global cognition. This indicates that changes in global cognition are related to local, rather than large-scale network connectivity. We would expect higher level cognitive functions, such as global cognition, should require integration of information from different sources, and therefore benefit from global efficiency across a whole network.

To further explore the importance of precuneus in aging brain, a seed-based method was used to display the pattern of functional connectivity. The most straightforward way to examine the functional connections of a particular brain region is to correlate the resting-state time-series of the depicted brain region against the time-series of all other regions (Van Den Heuvel and Hulshoff Pol, 2010). The wide-spread connections seen by this method suggest the precuneus is a major association area which may subserve a variety of cognitive-behavioral functions, as has been previously suggested (Cavanna and Trimble, 2006). Clusters that our study depicted were not all included in DMN, but they participate in distinct functional networks which control high-level cognition. Several other studies have proposed that precuneus is connected to brain networks that differ from DMN (Cavanna and Trimble, 2006; Margulies et al., 2009; Utevsky et al., 2014). However, the main functional connectivity clusters were located in bilateral precuneus, which showed an extensive autocorrelation. These results also suggested that the precuneus functional connectivity is relatively stabilized. In addition, precuneus is one of the regions to be affected early in AD, and it is difficult to be certain that early AD pathology is not, at least, partially responsible for this change with aging.

In the present study, we also found age-related changes in cortical thickness. There was cortical atrophy with age in the bilateral medial prefrontal, cingulum extending to PCC, and right dorsolateral temporal cortex, regions that form nodes of the default network. We also found that two small cortical areas of left middle temporal and left supramarginal gyrus were thicker in older participants, which indicates the ability of some brain regions to compensate for cortical atrophy. Alterations of cortical thickness did not exhibit any significant associations with cognitive functions. In fact, the evidence for a relation between cortical atrophy and cognitive decline is equivocal in humans (Ziegler et al., 2010; Tuladhar et al., 2015; Pini et al., 2016), and our results are consistent with this.

Several limitations of our study should be noted. First, our sample size was modest for a normal aging study. However, it is worth noting that the participants were very old (aged 78+), which can bring some new insights into longitudinal changes of normal aging in old-aged group. Larger samples may be required to detect more subtle relationships involving age, FCS, and cognition. Second, we did not explicitly evaluate the reliability the resting-state functional connectivity measures. Selective attrition and test–retest effects may influence the results (Taris and Kompier, 2003). Selective attrition adds bias but is a reliability issue. Third, tasks that were used to obtain the executive function compound score cannot present “executive function” sufficiently, as the concept of “executive function” is broad, heterogeneous, and complex. Fourth, for longitudinal FCS changes, we did not control sex and age in the longitudinal analysis, and this would be our areas of future research. Fifth, the statistical analysis was not completely identical—we used the permutation test as statistical modal in Section “Analysis of Longitudinal Changes in FCS,” and parametric test (paired *t*-test) in other sections. Finally, we did not have PET imaging to investigate associations between amyloid deposition in the brain and abnormal changes in FCS. Previous studies have shown that a significant proportion of cognitive unimpaired older adults may present substantial amyloid deposition in brain (Aizenstein et al., 2008). In the future, effective multimodal neuroimaging research should be used to deeply explore normal aging mechanisms.

## Conclusion

In conclusion, normal aging was accompanied by altered FCS and cognitive performance. Reduced FCS may affect main brain networks and explain age-related cognitive decline with aging. However, there is some evidence of increases in FCS with age, possibly playing a role in neural strategies change or compensatory mechanisms during the aging process. FCS changes are detectable with normal aging, and measures of ΔFCS are correlated with Δglobal cognition in the left precuneus. Our characterization of aging effects on brain networks is of great value to help better understand the potential aging mechanisms.

## Data Availability Statement

The datasets used and analyzed are available to other researchers subject to review of the request by the Scientific Committee of the study and ethics approval. Data are moreover already shared in COSMIC consortia.

## Ethics Statement

The studies involving human participants were reviewed and approved by the Ethics Committees of the University of New South Wales and the South Eastern Sydney and Illawarra Area Health Service. The patients/participants provided their written informed consent to participate in this study. Written informed consent was obtained from the individual(s) for the publication of any potentially identifiable images or data included in this article.

## Author Contributions

TL, CD, and QL contributed to study design, data analyses, interpretation of the results, and manuscript writing. WW contributed to study design and interpretation of the results. HB, PS, and NK contributed to data collection and interpretation of the results. XC, AP, JJ, JC, and HN contributed to data analyses. All authors participated in manuscript revision and final approval.

## Conflict of Interest

The authors declare that the research was conducted in the absence of any commercial or financial relationships that could be construed as a potential conflict of interest.
