# Supplementary material for: Longitudinal Changes in Whole-Brain Functional Connectivity Strength Patterns and the Relationship With the Global Cognitive Decline in Older Adults
Source: Front Aging Neurosci. 2020 Mar 17;12:71. doi: 10.3389/fnagi.2020.00071 (PMC7090100; doi:10.3389/fnagi.2020.00071)
Supplement: Supplementary file 1 [file Data_Sheet_1.pdf]

Supplementary Material

1 Neuropsychological assessments in the study

Table S1: Neuropsychological test battery administered in the study

Cognitive Domain	Test
Attention/Processing speed	Digit Symbol-Coding (Wechsler, 1997a)<br>Trail Making Test (Strauss et al., 2006)
Executive function	Controlled Oral Word Association Test (FAS) (Strauss et al., 2006)<br>Trail Making Test (TMT) B (Strauss et al., 2006)
Language	Boston Naming Test – 30 items (Kaplan, 2001)<br>Semantic Fluency (Animals) (Strauss et al., 2006)
Visuo-spatial	Block Design (Wechsler, 1981)
Memory	Logical Memory Story A delayed recall (Wechsler, 1997b)<br>Rey Auditory Verbal Learning Test (Strauss et al., 2006)<br>Benton Visual Retention Test recognition (Benton et al., 1996)

Benton, A.L., Sivan, A.B., and Spreen, O. (1996). *Der Benton Test.* Bern: Huber.

Kaplan, E. (2001). *The Boston Naming Test.* Philadelphia: Lippincott Williams Wilkins.

Strauss, E., Sherman, E.M.S., and Spreen, O. (2006). *A Compendium of Neuropsychological Tests: Administration, Norms, and Commentary.* New York: Oxford University Press.

Wechsler, D. (1981). *WAIS-R manual.* New York: The Psychological Corporation.

Wechsler, D. (1997a). *WAIS-III: Wechsler adult intelligence scale.* Psychological Corporation San Antonio, TX.

Wechsler, D. (1997b). *Wechsler memory scale (WMS-III).* Psychological Corporation San Antonio, TX.

2 Supplementary Figure

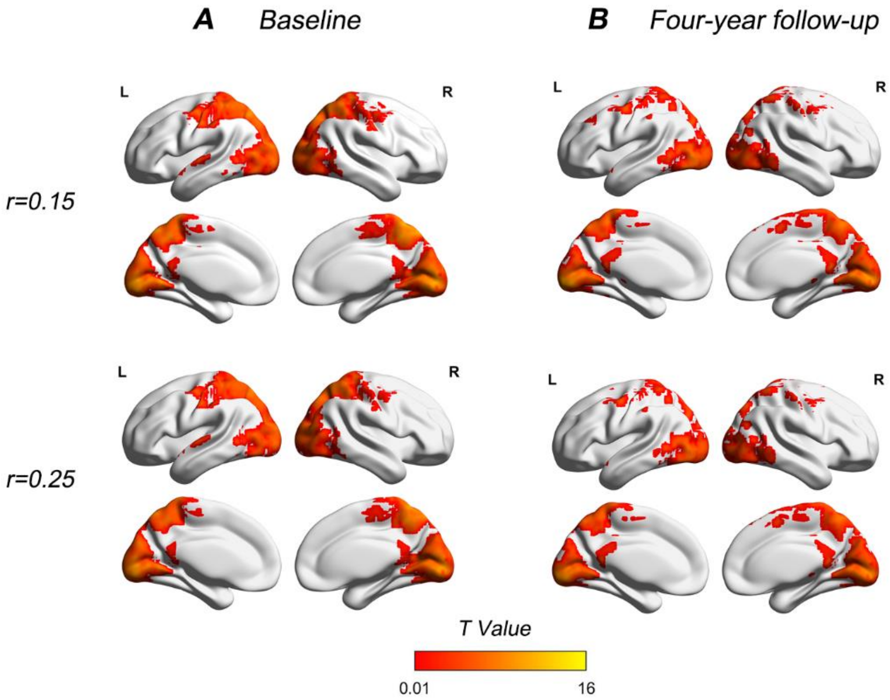

Figure S1. Spatial FCS patterns with different r threshold at two time points. (A) FCS patterns in baseline when r=0.15 (top), r=0.25 (bottom). (B) FCS patterns in four-year follow-up when r=0.15 (top), r=0.25 (bottom). Brain regions showing higher FCS were mainly located in precuneus, calcarine, inferior occipital, and inferior temporal. FCS maps with different r threshold showed similar distribution patterns. FCS, functional connectivity strength; T, statistical value; L, left bran; R, right brain.

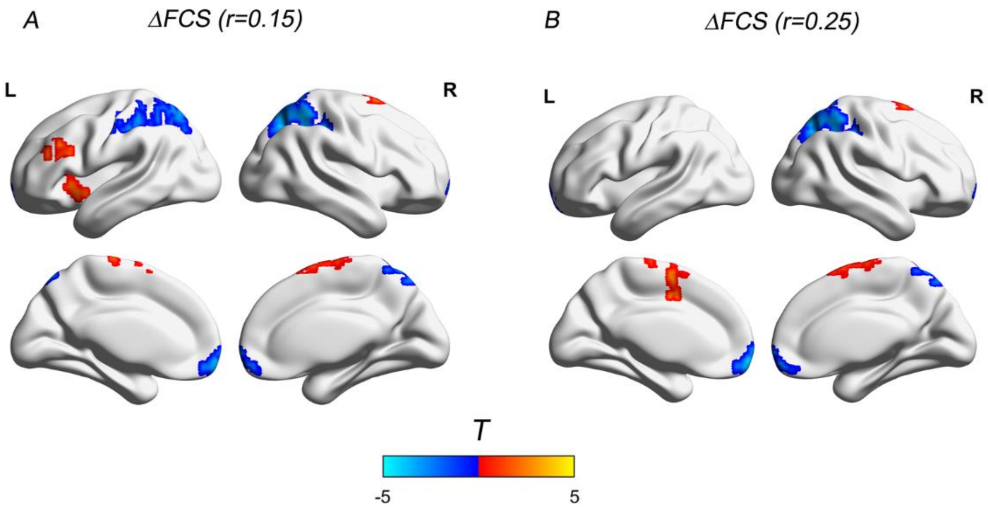

Figure S2. The longitudinal changes in FCS with different r threshold at two time points. (A) longitudinal changes in FCS with a threshold of r=0.15. (B) longitudinal changes in FCS with a threshold of r=0.25. With different r value, there were slightly differences in the ΔFCS, and most brain regions with ΔFCS were preserved. These maps were similar to the ΔFCS map when r=0.2. FCS, functional connectivity strength; ΔFCS, follow-up FCS minus baseline FCS; T, statistical value.

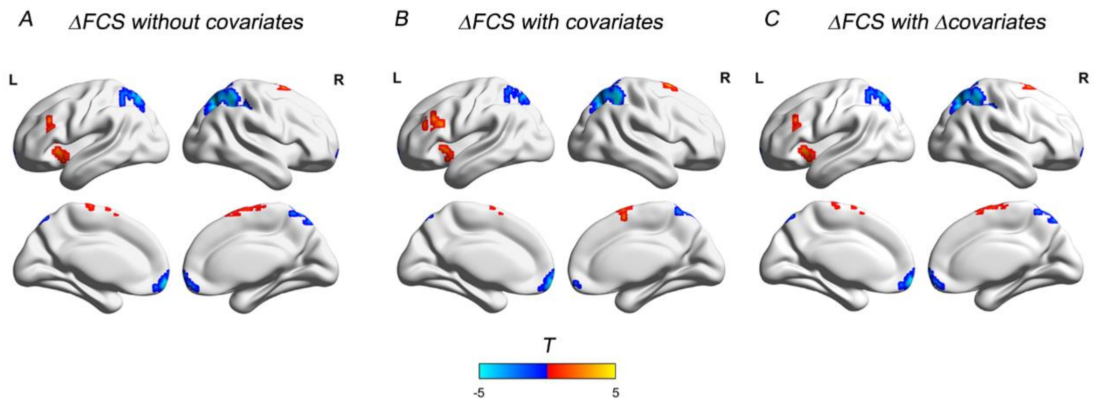

Figure S3. The longitudinal changes in FCS without/with grey matter volume as covariates. (A) longitudinal changes in FCS without grey matter volume as covariates. (B) longitudinal changes in FCS with two-timepoint grey matter volume as covariates. (C) longitudinal changes in FCS with Δcovariates (follow-up grey matter volume minus baseline grey matter volume). All results had same significantly altered regions located in left SMA, left insula, right superior parietal, left superior parietal and right medial frontal. FCS, functional connectivity strength; ΔFCS, follow-up FCS minus baseline FCS; Δcovariates, follow-up grey matter volume minus baseline grey matter volume; SMA, supplementary motor area; T, statistical value.

3 Seed-based analysis

The left precuneus (MNI coordinates -6 -66 54 mm), which showed robust relationship with Δglobal cognition, was used as the seed (number of voxels = 20). The main functional connectivity clusters were located in bilateral precuneus and right medial cingulum. The main functional connectivity clusters information was summarized in Table S2. The correlations at both baseline and 4-year follow-up showed a similar pattern (Figure S4).

Based on findings of the correlation relationship, we performed the seed analysis. Seed points were chosen from voxels with the significant relationship between ΔFCS and Δcognition. Seed-based analysis was performed to explore the connectivity pattern between BOLD signal fluctuations of the seed and BOLD signal fluctuations from all other voxels in the brain. Voxels with significant correlations were then selected (voxel-wise: minimum z-value> 3.29; cluster significance: $p < 0.001$, two-tailed GRF correction).

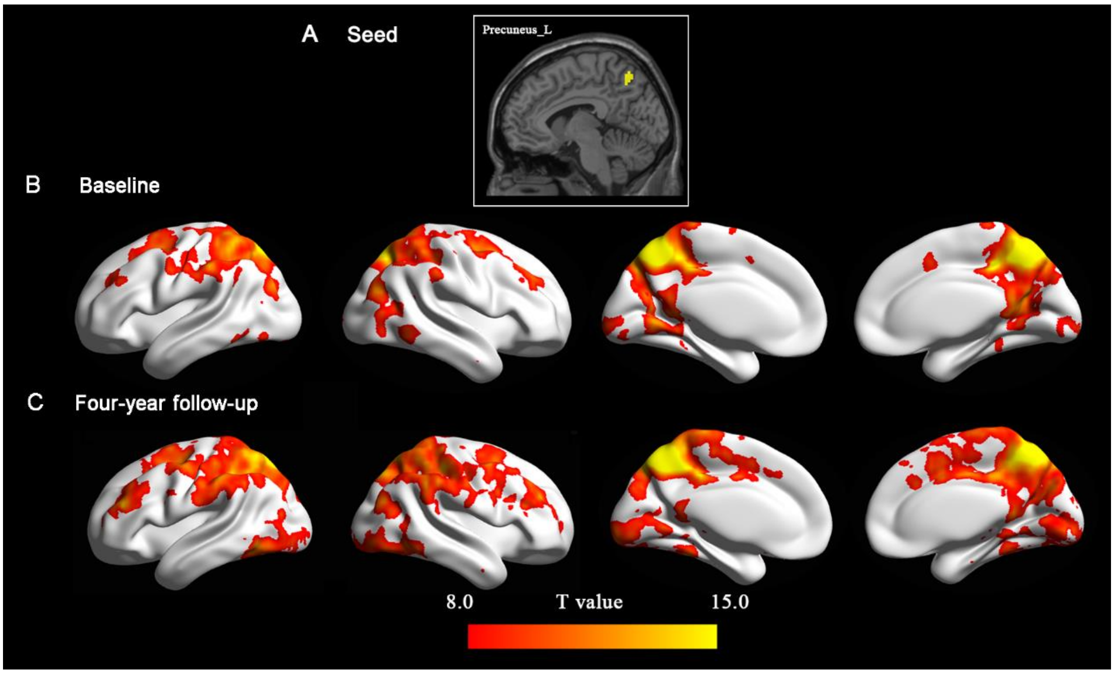

Figure S4. Cognition connectivity map generated with the seeds in left precuneus. (A) The seed located in left precuneus. (B) The seed-based analysis was applied to baseline scans. (C) The seed-based analysis was applied to four-year follow-up scans. Warm colors (red and yellow) represent positive functional connectivity.

Table S2: Seed-based analysis at two time points

	Brain regions	BA	Cluster size	Peak MNI coordinates, mm			T
				x	y	z	
**Baseline**	Left precuneus	7	41782	-6	-63	54	33.88
	Right precuneus	7	-	6	-63	54	24.72
	Right medial cingulum	23	-	12	-36	42	21.95
**4-year follow-up**	Left precuneus	7	43014	-6	-63	57	32.46
	Left precuneus	5	-	-3	-54	54	28.07
	Right precuneus	7	-	9	-63	54	23.82

BA, Brodmann's area; MNI, Montreal Neurological Institute;
T, statistical value of peak voxel;

4 References

Reuter, M., and Fischl, B. (2011). Avoiding asymmetry-induced bias in longitudinal image processing. *Neuroimage* 57**,** 19-21.

Reuter, M., Schmansky, N.J., Rosas, H.D., and Fischl, B. (2012). Within-subject template estimation for unbiased longitudinal image analysis. *Neuroimage* 61**,** 1402-1418.

Desikan, R.S., Segonne, F., Fischl, B., Quinn, B.T., Dickerson, B.C., Blacker, D., Buckner, R.L., Dale, A.M., Maguire, R.P., Hyman, B.T., Albert, M.S., and Killiany, R.J. (2006). An automated labeling system for subdividing the human cerebral cortex on MRI scans into gyral based regions of interest. *Neuroimage* 31**,** 968-980.
